# The RASA2/RASA3 ortholog RasGAP1 modulates obesity-linked phenotypes and is associated with leptin-analog signaling in *Drosophila*

**DOI:** 10.3389/fendo.2026.1773383

**Published:** 2026-05-20

**Authors:** Thiago C. Moulin, Michael J. Williams, Helgi B. Schiöth

**Affiliations:** 1 Department of Surgical Sciences, Uppsala University, Uppsala, Sweden; 2 Department of Pharmaceutical Biosciences, Uppsala University, Uppsala, Sweden; 3 Laboratory of Pharmaceutical Pharmacology, Latvian Institute of Organic Synthesis, Riga, Latvia

**Keywords:** energy homeostasis, feeding behavior, metabolism, neuroendocrinology, obesity, RASA2/3

## Abstract

**Introduction:**

Obesity arises from the interplay between genetic predisposition, metabolic signaling, and neural circuits that regulate feeding and energy expenditure. Large-scale association studies repeatedly implicate regulators of Ras/Rap GTPase signaling in adiposity and metabolic risk, but the mechanisms linking these intracellular switch modules to neural control of energy balance remain unclear.

**Methods:**

We used a translational approach centered on Drosophila RasGAP1, the closest ortholog of mammalian RASA2/3. Pan-neuronal RasGAP1 knockdown was assessed for effects on locomotor behavior, feeding interactions, systemic metabolic markers, expression of the leptin analog unpaired 1 (upd1), and protein-interaction networks. We then extended these findings to humans using protein-network and phenome-wide association analyses focused on RASA2/3-related pathways.

**Results:**

Pan-neuronal RasGAP1 knockdown shifted behavior toward an obesity-like phenotype, combining reduced locomotor output with increased feeding interactions. This was accompanied by elevated lipid storage, increased circulating sugars, and reduced expression of upd1. Protein-interactome mapping positioned RasGAP1 within a connected signaling neighborhood linking Ras signaling with cytokine pathways relevant to feeding control. In humans, protein-network and phenome-wide association analyses converged on a KRAS-centered pathway in which RASA2/3, KRAS, and LEP were consistently associated with fat mass, BMI, and lipid dysregulation.

**Discussion:**

These findings support RasGAP1/RASA2/3 as a candidate conserved neuro-metabolic regulator. More broadly, they provide hypothesis-generating evidence that RasGAP dysfunction may bias neural and metabolic control systems toward adiposity-linked phenotypes.

## Introduction

Obesity is a complex disorder emerging from interactions between environmental exposures, metabolic state, and genetically encoded regulatory pathways. Both human genetic studies and mechanistic analyses highlight the importance of signaling pathways that translate nutrient cues into behavioral and metabolic outputs. Among these, the Ras/Rap small GTPase systems act as central hubs that integrate growth factor signaling, neuronal activity, and metabolic regulation. Members of the Ras superfamily of small monomeric GTP-binding proteins regulate a wide spectrum of biological processes, from transmembrane signal transduction to cytoskeletal dynamics and vesicular trafficking (1). These proteins cycle between an active GTP-bound and inactive GDP-bound state, a transition controlled by guanine nucleotide exchange factors (GEFs) and GTPase-activating proteins (GAPs), the latter accelerating Ras intrinsic GTP hydrolysis (2).

Among GAP1-family members, RASA2 (RAS p21 protein activator 2) and RASA3 (RAS p21 protein activator 3) play a critical role in regulating RAS and RAP1 signaling and thereby modulating proliferation, adhesion, and metabolic pathways (3, 4). Human studies further suggest that these proteins are functionally linked to metabolic status. For instance, DNA methylation at RASA3 associates with dietary fiber intake, visceral adiposity, and inflammation (5). Moreover, the single‐nucleotide polymorphism (SNP) rs16851483 in RASA2 was reported as associated with increased BMI in several genome-wide association studies (GWAS) (6–8). Yet, despite emerging evidence connecting RASA2/3 to obesity-related traits, the mechanisms through which they regulate energy balance remain unclear.

In *Drosophila*, the Ras GTPase activating protein 1 (RasGAP1, also termed Gap1; FBgn0004390) is the predicted ortholog of mammalian RASA2/3 and participates in multiple Ras-dependent pathways. Early developmental studies identified *Drosophila* RasGAP1 as a negative regulator of the Sevenless receptor pathway, where it downregulates Ras1 and prevents inappropriate R7 photoreceptor fate (9). Ectopic RasGAP1 expression in imaginal discs suppresses the signaling of the Fibroblast Growth Factor receptor homologs Breathless and Heartless, leading to reduced wing size (10, 11). RasGAP1 also modulates the signaling of the EGF receptor homolog during embryogenesis (12) and contributes to EGFR-dependent wing morphology (13). Its involvement in Pvr-mediated (PDGF receptor homolog) control of cell size (14) and broad participation in signaling pathways mediated by Ras and receptor tyrosine kinases (11, 15) further emphasizes its extensive regulatory role.

Moreover, public transcriptomic resources support detectable expression of RasGAP1 in the adult *Drosophila* brain, as shown by the FlyAtlas dataset (16). However, the function of neuronal RasGAP1 in physiological and behavioral regulation, as well as the pathways linking RasGAP1 deficiency to metabolic phenotypes, remains unresolved. Importantly, metabolic homeostasis in *Drosophila* is strongly shaped by neuroendocrine circuits, including insulin-producing cells and central neuromodulatory pathways that coordinate feeding decisions with systemic carbohydrate and lipid balance (17, 18). Consistent with this organization, discrete neuronal populations can exert hormone-like control over feeding drive and metabolic state, for example, via NPF-dependent (NPY homolog) satiety circuitry and peptide modulators that influence triglyceride levels (19, 20). In addition, the leptin analog upd1 is produced in the brain and acts through a conserved neuroendocrine circuit to regulate obesity-linked behaviors in flies (21, 22), supporting that central signals can functionally parallel mammalian endocrine regulators even when their tissue of origin differs.

Here, we show that pan-neuronal knockdown of RasGAP1 reduces movement, increases food intake, and elevates triacylglyceride and glucose levels, with more modest directional effects on trehalose measures, features consistent with an obese-like metabolic state. We further observe that upd1 expression is reduced when RasGAP1 is suppressed, identifying a candidate link between the RASA2/3 ortholog and the conserved leptin-analog circuit (21). Finally, by combining STRING-based network analyses with PheWAS results from GWAS Atlas, we outline a translational pathway hypothesis in which RasGAP proteins interface with a KRAS-centered signaling pathway that is consistently associated with adiposity and metabolic phenotypes in humans. This cross-species convergence suggests Ras85D/KRAS as a candidate intermediary linking RasGAP1 perturbation to leptin-analog-related metabolic outcomes.

## Methods

### Identification of RasGAP orthologs, multiple sequence alignment, and phylogenetic reconstruction

Putative orthologs of the RasGAP family were identified using the DRSC Integrative Ortholog Prediction Tool (DIOPT) (23), with *Drosophila melanogaster* RasGAP1 as the query. For each representative species (*Homo sapiens, Mus musculus, Gallus gallus, Danio rerio, Caenorhabditis elegans*), all predicted orthologs and closely related paralogs from the RASAL/RASA subfamilies were retrieved. Corresponding protein sequences were downloaded in FASTA format from UniProt (24). Protein sequences were imported into Jalview (25), where a multiple sequence alignment was generated using the built-in alignment tool with default parameters. The alignment was inspected manually and minimally trimmed to remove poorly aligned N-terminal tails while preserving all conserved domains (C2, PH, and RasGAP domains). The finalized alignment dataset is provided as **Supplementary File 1**.

Phylogenetic reconstruction was performed using IQ-TREE 3 (26) with maximum-likelihood inference. ModelFinder was used to automatically determine the best-fitting amino acid substitution model. Branch support was assessed using 1,000 ultrafast bootstrap replicates (UFBoot) and 1,000 SH-aLRT tests. The resulting Newick tree (**Supplementary File 2**) was visualized and annotated using iTOL (27).

### Fly husbandry, genotypes, and handling

Fly stocks were maintained at 25 °C with ~60% relative humidity in a 12:12 h light:dark cycle (lights on at 08:00). Unless otherwise stated, flies were reared on Jazz-Mix™ *Drosophila* food (Thermo Fisher Scientific, Göteborg, Sweden) supplemented with 8.3% yeast. Stocks were maintained in bottles with 40–70 flies per culture, flipped weekly into fresh food, and discarded after one month.

The following strains were obtained from the Bloomington *Drosophila* Stock Center (BDSC, Bloomington, IN, USA): (i) elav^C155^-GAL4, a pan-neuronal driver (BDSC #458; genotype: *P{w[+mW.hs]=GawB}elav[C155])*; (ii) UAS-RasGAP1^RNAi^ (BDSC #41830; genotype: *y[1] v[1]; P{y[+t7.7] v[+t1.8]=TRiP.GL01258}attP2*; which expresses dsRNA targeting RasGAP1 under UAS control); and (iii) w^1118^, used as a wild-type control. Knockdown flies were generated by crossing virgin females elav^C155^-GAL4 with males of the UAS-RNAi line, yielding neuronal knockdown in the F1 progeny (elav-GAL4>RasGAP1^RNAi^). Two control groups were generated in parallel: (i) elav^C155^-GAL4 females × w^1118^ males (elav-GAL4>w^1118^) and (ii) w^1118^ females × UAS-RasGAP1^RNAi^ males (w^1118^>RasGAP1^RNAi^). Crosses were typically set with ~30 virgin females and ~15 males per bottle. Parental flies were removed after three days. F1 males were collected every 1–2 days after eclosion and aged in same-sex vials (maximum 30 flies per vial) at 29 °C under the same light:dark cycle for 5–6 days prior to testing. All experiments were therefore performed on adult male flies. Mating status was not experimentally controlled as a variable. Because elav-GAL4 was used constitutively, RasGAP1 knockdown was present from development onward and the present design does not distinguish developmental from adult-specific neuronal effects.

### Feeding behavior (FlyPAD)

Feeding behavior was quantified using the FlyPAD system, a capacitive-based platform that records proboscis-food interactions, following stabilized protocols (28). Briefly, individual flies were transferred by mouth aspiration into arenas containing two food channels, one filled with 4-5 µL of standard liquid food and the other left empty. The primary outcome measure was the number of sips, defined as a single proboscis-food interaction, which is shown to correlate with food intake volume (29, 30). Assays were conducted for one hour between 08:00 and 11:00, with control and knockdown groups tested in parallel under identical conditions. Flies that remained completely immobile for the duration of the assay were excluded from analysis. The experiments were performed using the 2018 FlyPAD version. The behavioral data included in this study were obtained from two independent experimental batches.

### General locomotor activity (DAMS)

General locomotor activity was evaluated using the *Drosophila* Activity Monitor System (DAMS; TriKinetics Inc., Waltham, MA, USA) as previously reported (31). Flies were briefly anesthetized with CO_2_ and individually transferred into horizontal plastic tubes sealed at one end with standard fly food and at the other with a cotton plug to allow ventilation. An infrared beam at the midpoint of each tube recorded every crossing as an activity count. Activity was recorded continuously over 3 days under a 12:12 h light:dark cycle, starting at lights-on. To allow acclimation, the first 24 h of data were excluded from analysis. Raw data were exported as CSV files using DamFileScan (TriKinetics). Locomotor activity was defined as the number of infrared beam crossings per unit time, and activity traces were summarized in 30-minute bins for analysis and visualization. Flies that remained completely immobile for the duration of the assay were considered dead and excluded from analysis. The DAMS data included here were obtained from two independent experimental batches. Both batches showed the same direction of effect, and data were unified for the final statistical analysis.

### Triacylglyceride and carbohydrate measurements

Whole-fly triacylglyceride (TAG) content was quantified under fed conditions using the same procedures and reagents as previously described in our laboratory protocol (32). Each TAG biological sample consisted of pooled adult male flies. Briefly, 25 flies were homogenized in 100 µL PBST (1× PBS, 0.5% Tween 20), incubated at 70 °C for 5 min, and clarified by centrifugation. Free glycerol reagent was added first and absorbance was measured at 540 nm, after which triglyceride reagent was added and the final absorbance was measured at 540 nm. TAG concentrations were determined from a glycerol standard curve. Total protein was quantified from the same lysates, and TAG values were normalized to protein content and reported as µg TAG per mg protein, consistent with the figure axis. This assay was used as a whole-organism metabolic readout and was not intended to localize lipid accumulation to a specific tissue.

Circulating glucose and trehalose, as well as whole-body trehalose, were also measured. Adult flies were frozen at −80 °C overnight prior to processing. Each biological sample consisted of pooled adult male flies (10–15 flies; total sample weight 10–15 mg). To obtain the circulating fraction, frozen flies were decapitated and placed in PBS (pH 7.4) using 5 µL PBS per mg fly mass, followed by centrifugation (3000 rpm for 6 min at 4 °C). The collected circulating fraction was heat-treated at 70 °C for 5 min, cooled on ice, and centrifuged (15 min, 16,000 × g, 4 °C) to precipitate denatured proteins. Circulating glucose was measured directly from untreated aliquots of this supernatant, whereas circulating trehalose was measured in matched aliquots after enzymatic conversion to glucose using porcine kidney trehalase (Sigma, T8778) incubated overnight at 37 °C. To obtain trehalose-specific values, the glucose concentration measured in untreated aliquots, corresponding to free glucose, was subtracted from the glucose concentration measured after trehalase treatment, corresponding to free glucose plus trehalose-derived glucose. The resulting trehalose-derived glucose values were then converted to trehalose equivalents and expressed as circulating trehalose.

For whole-body trehalose, the remaining bodies were homogenized in PBS using 10 µL PBS per mg fly mass. Pellets were homogenized using a plastic pestle, centrifuged (15 min, 16,000 × g, 4 °C), and the resulting supernatant was collected for measurement. Trehalose in body supernatants was converted to glucose by trehalase incubation overnight at 37 °C prior to glucose quantification. As for the circulating fraction, whole-body trehalose values were calculated by subtracting the glucose concentration measured before trehalase treatment from the glucose concentration measured after trehalase treatment, and the resulting values were converted to trehalose equivalents.

Glucose levels were quantified using the Liquick Cor-Glucose diagnostic kit (Cormay), based on glucose oxidase/peroxidase chemistry. Absorbance was measured at 492 nm on a microplate spectrophotometer and converted to concentration using a calibration curve generated from serial dilutions of a glucose standard. Concentrations were then back-calculated to the original substrate and expressed as mM for circulating glucose/trehalose or µg per mg wet weight for whole-body trehalose, matching the units reported in the figure. As the circulating fraction was obtained from decapitated frozen flies, these readouts are interpreted here as circulating sugar measures; however, some contribution from tissue-derived contents cannot be fully excluded.

### Quantitative RT–PCR

Gene expression was quantified using quantitative RT–PCR in heads of RasGAP1 pan-neuronal knockdown flies and matched controls. Each biological sample consisted of 25 adult male heads. Heads were homogenized in 460 µL Trizol, followed by addition of 160 µL chloroform. Samples were centrifuged for 12 min at 14,000 rpm at 4 °C, and at least 200 µL of the upper aqueous phase was transferred to a new tube. RNA was precipitated with 450 µL isopropanol, incubated at subzero temperature to increase yield, centrifuged for 15 min at 14,000 rpm at 4 °C, washed three times in ice-cold 75% ethanol, air-dried for 20 min, and resuspended in 20 µL DEPC-treated water. RNA concentration and purity were measured using a NanoDrop Multiskan Go spectrophotometer (Thermo Scientific). For cDNA synthesis, the High-Capacity RNA-to-cDNA kit (Thermo Scientific) was used according to the manufacturer’s protocol, yielding a 20 µL reaction with a final cDNA concentration of 100 ng/µL. cDNA was subsequently diluted 1:30 in Milli-Q H_2_O prior to qPCR.

Each reaction contained 17 µL of master mix and 3 µL of diluted cDNA in a 96-well plate. The master mix consisted of 11.52 µL Milli-Q H_2_O, 3.6 µL Taq buffer, 0.20 µL 80 mM dNTPs, 0.10 µL 100 µM forward/reverse primer mix, 1.00 µL DMSO, 0.50 µL SYBR Green, and 0.08 µL Taq polymerase. All samples were run in triplicate, and each plate included three no-template controls. Primers used were as follows. RasGAP1: Forward 5′-AAATTGGCGAGGCAAAGAATCT-3′, Reverse 5′-CGCGGAATCTTGAACTGGTG-3′. upd1: Forward 5′-CAGCGCACGTGAAATAGCAT-3′, Reverse 5′-CGAGTCCTGAGGTAAGGGGA-3′. RpL32 (housekeeping gene): Forward 5′-AGCATACAGGCCCAAGATCG-3′, Reverse 5′-TGTTGTCGATACCCTTGGGC-3′.

Ct values were analyzed using the ΔΔCt method (33). Samples with primer efficiencies deviating markedly from the plate mean were excluded. Triplicates with Ct differences greater than 0.5 cycles were partially or fully removed depending on individual replicate efficiency. Final ΔΔCt values were computed using RpL32 as the normalization reference. For the present manuscript, qPCR comparisons were made between elav-GAL4>RasGAP1^RNAi^ and the driver control elav-GAL4>w1118.

### STRING protein–protein interaction network analysis

Protein–protein interaction networks were generated using the STRING database (v12.0) via the web interface (34). For the *Drosophila* network, RasGAP1 and upd1 were used as query proteins with the organism set to *Drosophila melanogaster*. For the human network, RASA2, RASA3, KRAS, and LEP were used as query proteins with the organism set to *Homo sapiens*. Networks were constructed as full STRING networks using a medium-confidence minimum interaction threshold (STRING combined confidence score). Each network was expanded by adding up to five first-shell interactors to recover the local signaling neighborhood around the query set. Networks were visualized in evidence view with all evidence channels enabled, so that edge colors reflect the supporting sources, including curated databases, experimental evidence, co-expression, predicted interactions (gene neighborhood and co-occurrence), and text mining. Final network images were exported directly from STRING for figure preparation. Importantly, these analyses were used to generate mechanistic hypotheses rather than to establish direct causal relationships.

### PheWAS analysis of human metabolic traits

Human genetic associations were evaluated using the GWAS Atlas database (accessed 28 November 2025). For each gene of interest (RASA2, RASA3, KRAS, and LEP), we retrieved all available PheWAS results and restricted the analysis to traits classified as metabolic or anthropometric outcomes. After filtering, a total of 1,259 GWAS were considered, including studies in which the queried genes or variants were not directly tested. Only associations that surpassed the Bonferroni-corrected significance threshold (p < 3.97 × 10^−5^) were retained for downstream interpretation. For visualization, each retained trait was plotted by -log_10_(p-value), and the number of significant traits per gene was summarized in the corresponding figure panels. Traits were annotated and grouped according to their physiological domains, with emphasis on the most significant associations. As with STRING, these analyses were used here to provide translational context rather than mechanistic proof.

### Statistical analysis

All data were analyzed using GraphPad Prism (v. 10.4; GraphPad Software, San Diego, CA, USA). For DAMS locomotor activity time-course data (30 min bins across the day), genotype effects were assessed using a two-way repeated-measures ANOVA with factors Time and Genotype, including the Time × Genotype interaction. Summary measures (e.g., 24 h total activity, averaged per 30 min bin) and FlyPAD feeding outcomes (e.g., number of sips) were analyzed using one-way ANOVA followed by Holm–Šídák multiple-comparisons tests. For qPCR experiments, ΔΔCt values were compared between elav-GAL4>RasGAP1^RNAi^ and the driver control elav-GAL4>w^1118^ using an unpaired two-tailed t-test. Data are reported as mean ± SEM, and adjusted p-values are shown in the corresponding figures. For transparency, we provide a spreadsheet with means, SD and sample size for all results described in our study, available on **Supplementary File 3**.

## Results

### *Drosophila* RasGAP1 is an evolutionarily conserved ortholog of RASA2 and RASA3

To identify the closest vertebrate counterparts of *Drosophila* RasGAP1, we queried the DRSC Integrative Ortholog Prediction Tool (DIOPT) (23) using RasGAP1 as input. Among all predicted human gene matches, RASA3 and RASA2 consistently received the highest overall orthology scores, whereas other RasGAP family members (RASAL1, RASAL2, RASA4) returned lower or moderate scores. This suggested that RasGAP1 most likely corresponds to the ancestral gene that gave rise to the RASA2/3 branch in vertebrates.

**Figure 1:**
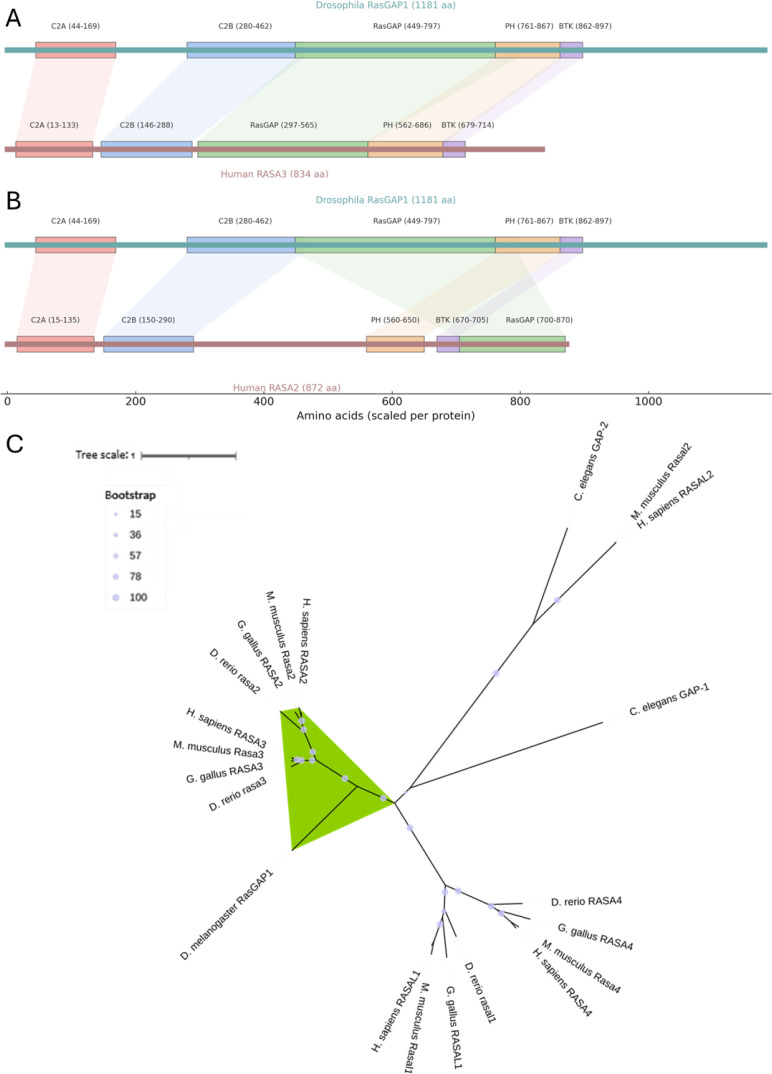
Comparative domain architecture and phylogenetic placement of *Drosophila* RasGAP1. **(A, B)** Domain architecture comparison of *Drosophila* RasGAP1 with human RASA3 **(A)** and RASA2 **(B)**. Schematic protein illustrations demonstrate the relative positions of conserved domains, including the C2A, C2B, RasGAP, PH, and BTK domains. Each domain is scaled to its position within the full-length protein, and colored to highlight homologous regions across species. The overlays emphasize the high positional and structural conservation of the RasGAP, C2, PH, and BTK domains between *Drosphila melanogaster* RasGAP1 and human RASA2/3, despite lineage-specific insertions and differences in total protein length. These conserved domain organizations support functional orthology. **(C)** Maximum-likelihood phylogeny of RasGAP family proteins across representative metazoans. The tree includes RasGAP paralogs from *Homo sapiens, Mus musculus, Gallus gallus, Danio rerio, Drosophila melanogaster*, and *Caenorhabditis elegans*. Phylogenetic inference was performed using IQ-TREE 3. Bootstrap support is shown as node bubbles (legend at left). The RasGAP1/RASA2/3 clade is highlighted in green.

To examine structural conservation directly, we compared the domain architectures of RasGAP1 with human RASA3 and RASA2 (**Figures 1A, B**). Despite differences in total sequence length, RasGAP1 retained a highly conserved arrangement of C2, RasGAP, PH, and BTK domains, with broadly similar spacing and boundaries. The preservation of this multi-domain organization across insects and mammals supports functional continuity between RasGAP1 and RASA2/3. To test this inference phylogenetically, we reconstructed a maximum-likelihood tree including all RasGAP paralogs returned by the orthology search of *D. melanogaster* RasGAP1, together with their representative homologs from *Homo sapiens*, *Mus musculus*, *Gallus gallus*, *Danio rerio*, and *Caenorhabditis elegans* (**Figure 1C**). In this broader context, RasGAP1 did not group with the RASAL1/2 or RASA4 subfamilies, even though these clades include both vertebrate and non-vertebrate representatives such as *C. elegans* GAP-2. Instead, RasGAP1 consistently formed the invertebrate sister lineage to the RASA2/3 clade, with strong statistical support (97–100% SH-aLRT and UFBoot). This topology indicates a many-to-one orthology, in which RasGAP1 in insects may correspond to the ancestral gene that later duplicated in vertebrates to give rise to RASA2 and RASA3.

### Pan-neuronal knockdown of RasGAP1 reduces activity and increases food intake

**Figure 2:**
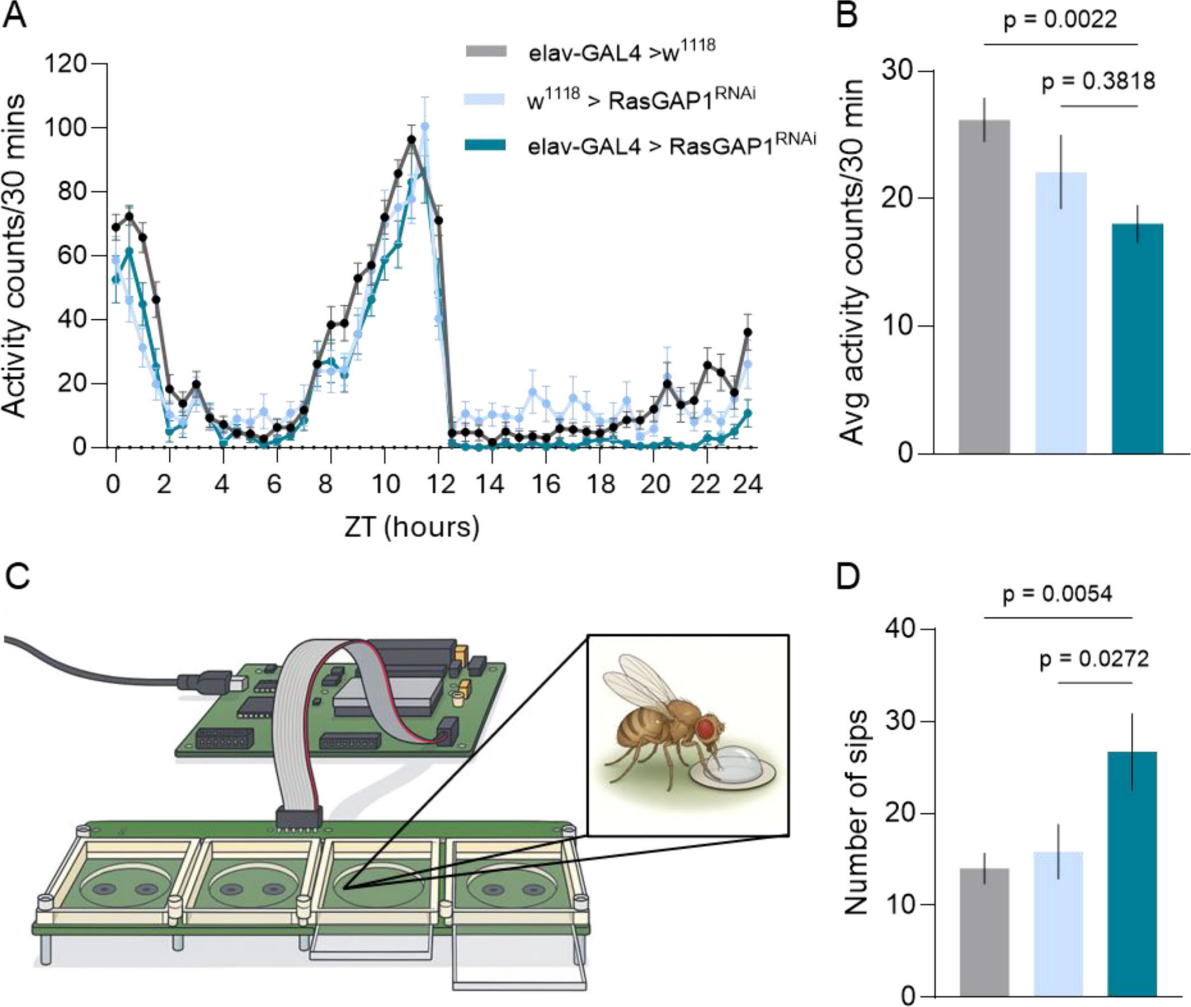
Pan-neuronal RasGAP1 knockdown reduces locomotor activity and increases feeding interactions. **(A)** Locomotor activity counts across 24 h under a 12:12h light-dark cycle, plotted in 30 min bins. Activity time series was analyzed by two-way repeated-measures ANOVA, revealing significant effects of Time and Time × Genotype (both p < 0.0001). **(B)** Total 24 h activity, expressed as the mean activity per 30 min across 24 h. Data were obtained from two independent experimental batches. **(C)** Schematic of the FlyPAD setup used to quantify proboscis contacts with liquid food. **(D)** Total number of sips recorded during the FlyPAD assay. FlyPAD data were obtained from two independent experimental batches. Data are represented as mean ± SEM. **(B, D)** Were analyzed by one-way ANOVA followed by Holm–Šídák multiple-comparisons test; adjusted p-values shown in the figure. For **(B)**, sample sizes were n = 24 for elav-GAL4>w^1118^, n = 31 for w^1118^>RasGAP1^RNAi^, and n = 10 for elav-GAL4>RasGAP1^RNAi^. For **(D)**, sample sizes were n = 55 for elav-GAL4>w^1118^, n = 31 for w^1118^>RasGAP1^RNAi^, and n = 52 for elav-GAL4>RasGAP1^RNAi^.

Having established that *Drosophila* RasGAP1 is the closest ortholog of mammalian RASA2/3, we next asked whether neuronal RasGAP1 suppression is sufficient to alter behavioral outputs relevant to energy balance. We therefore quantified daily locomotor activity rhythms and feeding interactions in elav-GAL4>RasGAP1^RNAi^ flies relative to the genetic controls elav-GAL4>w^1118^ and w^1118^>RasGAP1^RNAi^ (**Figure 2**).

Across the 24-hour cycle, pan-neuronal RasGAP1 knockdown produced a distinct activity profile compared with controls (**Figure 2A**). Two-way repeated-measures ANOVA detected significant effects of Time and Time × Genotype (both *p* < 0.0001), indicating that genotype-dependent differences varied across the day. The difference was not uniform across the full 24 h profile and was most evident during the 16–24 h interval, corresponding to the later part of the dark phase and the transition to the next lights-on period. Consistent with the time-course, overall locomotor output quantified as daily total activity (mean activity per 30 min across 24 h) was reduced in elav-GAL4>RasGAP1^RNAi^ (**Figure 2B**), although this summary effect was more robust relative to the driver control than to the UAS control and should therefore be interpreted cautiously.

Finally, to assess whether reduced activity was accompanied by altered feeding drive, we measured food interactions using the FlyPAD assay (**Figure 2C**). Despite reduced locomotor activity, elav-GAL4>RasGAP1^RNAi^ flies exhibited an increased number of sips compared with controls (**Figure 2D**), indicating elevated feeding interactions in this genotype.

### Pan-neuronal knockdown of RasGAP1 induces an obesity-like metabolic state

**Figure 3:**
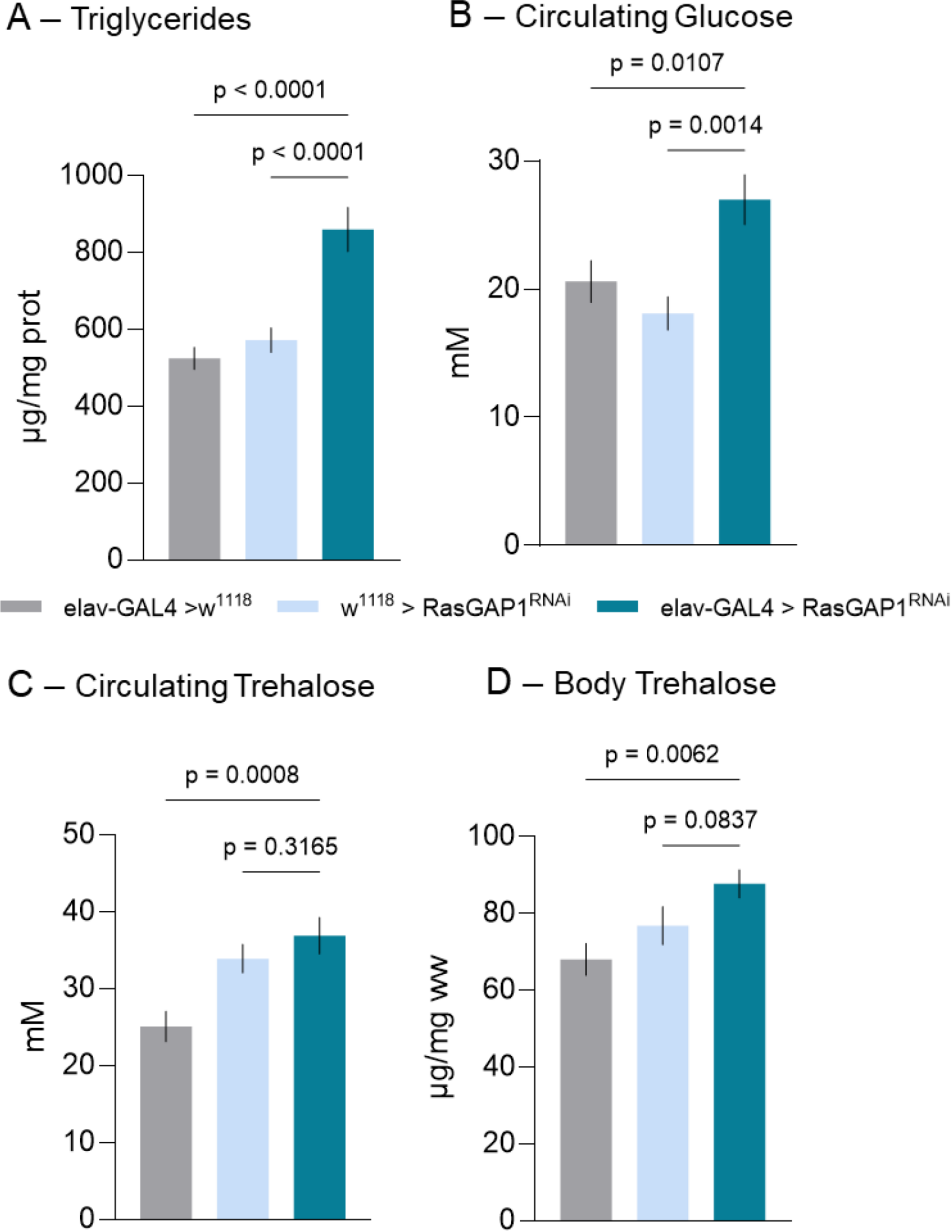
Pan-neuronal knockdown of RasGAP1 elevates lipid and carbohydrate levels. **(A)** Whole-body triacylglyceride (TAG) levels (µg/mg protein). **(B)** Circulating glucose levels (mM). **(C)** Circulating trehalose levels (mM). **(D)** Whole-body trehalose content (µg/mg wet weight). Bars represent mean ± SEM. Statistical significance was assessed by one-way ANOVA followed by Holm-Šídák multiple-comparisons test, n=10 replicates per group; TAG samples were measured from pooled biological samples of 25 adult male flies per replicate, and circulating/body carbohydrate samples were measured from pooled biological samples of 10–15 adult male flies per replicate; adjusted p-values are reported in the figure.

Motivated by the established role of *Drosophila* neuroendocrine circuits in coordinating whole-body carbohydrate and lipid homeostasis, we asked whether neuronal RasGAP1 suppression and the accompanying behavioral phenotypes extend to systemic metabolism. For this, we quantified lipid and carbohydrate parameters in elav-GAL4>RasGAP1^RNAi^ flies and compared them with the genetic controls elav-GAL4>w^1118^ and w^1118^>RasGAP1^RNAi^ (**Figure 3**).

We observed that pan-neuronal knockdown of RasGAP1 led to a marked increase in triacylglyceride levels relative to both control genotypes (**Figure 3A**), indicating enhanced lipid accumulation. In parallel, circulating glucose levels were elevated in elav-GAL4>RasGAP1^RNAi^ flies (**Figure 3B**), suggesting altered carbohydrate homeostasis. Moreover, given that trehalose represents the principal circulating sugar in insects, we next examined trehalose levels. Trehalose measures showed a directional increase in the knockdown group, particularly relative to the driver control (**Figures 3C, D**), but these differences were not significant relative to both parental controls and should therefore be interpreted cautiously. Together, these results support an obesity-like or adiposity-linked metabolic shift after pan-neuronal RasGAP1 knockdown, driven primarily by increased TAG and circulating glucose, while trehalose-related changes should be interpreted cautiously.

### RasGAP1 suppression is associate with leptin-analog pathways

**Figure 4:**
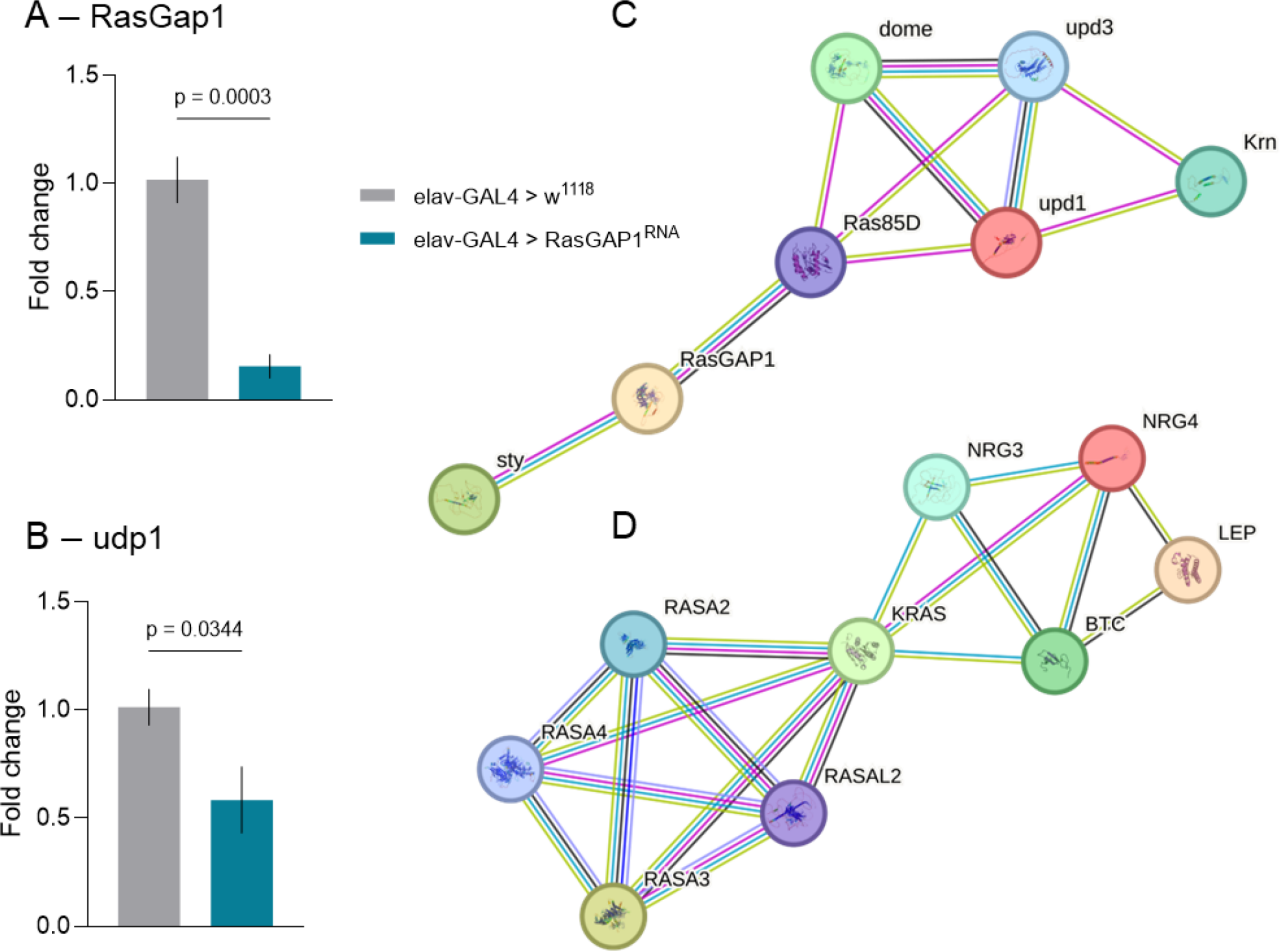
qPCR validation and STRING interaction networks for *Drosophila* and human orthologs. **(A, B)** Relative expression of RasGAP1 and upd1, quantified by qPCR. Both genes show significant downregulation compared with the elav-GAL4>w1118 driver control (ΔΔCt method). Bars represent mean ± SEM. Statistical analyses by unpaired t tests; n = 5 control samples and n = 4 knockdown samples, each consisting of 25 heads, with each sample run in technical triplicate. **(C)** STRING protein–protein interaction network for *Drosophila*, generated using RasGAP1 and upd1 as input. The analysis recovers a connected module including Ras85D, sty, dome, upd3, and Krn, consistent with predicted interactions within JAK/STAT and Ras signaling pathways. **(D)** STRING network for the human orthologs RASA2, RASA3, KRAS, and LEP. The resulting network shows interactions among RASA2, RASA3, KRAS, and associated partners (NRG3, NRG4, BTC), positioning these proteins within a shared growth-factor- and metabolism-related signaling environment. Edges represent combined confidence scores integrating curated databases (blue), experimental data (purple), co-expression (black), interaction from prediction gene neighborhood and co-occurrence (green/dark blue), and text mining (yellow). Adapted from a STRING-generated protein interaction network using the authors’ selected input proteins. The figure was formatted for presentation in the manuscript.

To characterize how neuronal RasGAP1 depletion influences signaling components linked to *Drosophila*’s behavioral and metabolic phenotypes, we quantified gene expression in heads of elav-GAL4>RasGAP1^RNAi^ flies. As expected, RasGAP1 levels were strongly reduced when compared to the elav-GAL4>w^1118^ controls (**Figure 4A**). Moreover, we measured upd1, the functional leptin analog (21), as the phenotypes observed in this model suggest that altered upd1 signaling might contribute to the obesity-related traits. Although upd1 is produced in the *Drosophila* brain rather than adipose tissue, it fulfills the leptin-like role of regulating feeding and weight through central nutrient-responsive circuits (21). Analysis by qPCR showed that upd1 was likewise downregulated in these flies (**Figure 4B**). Importantly, because this qPCR comparison was performed relative to the driver control and did not include the UAS-RNAi control, the upd1 result should be interpreted as candidate-pathway evidence rather than definitive proof of genotype-specific pathway regulation.

To contextualize these transcriptional changes, we constructed STRING interaction networks. In *Drosophila*, RasGAP1 and upd1 formed a coherent module with Ras85D, sty, dome, upd3, and Krn (**Figure 4C**), linking Ras/MAPK signaling with JAK-STAT cytokine pathways. This organization indicates that neuronal RasGAP1 suppression perturbs a signaling environment that integrates growth-factor and cytokine cues relevant to feeding behavior and metabolic control.

We next examined whether the human orthologs RASA2, RASA3, KRAS, and LEP participate in a comparable signaling neighborhood. The human STRING network revealed that these proteins cluster with NRG3, NRG4, and BTC (**Figure 4D**), placing RASA2/3 and KRAS within a shared growth-factor– and metabolism-related context. Although the specific receptors differ between species, the organization around KRAS and its GAPs suggests a conserved structural framework through which leptin-analog pathways interface with Ras signaling.

### RASA 2, RASA 3, KRAS, and LEP are associated with fat mass, BMI, and metabolic disorders

**Figure 5:**
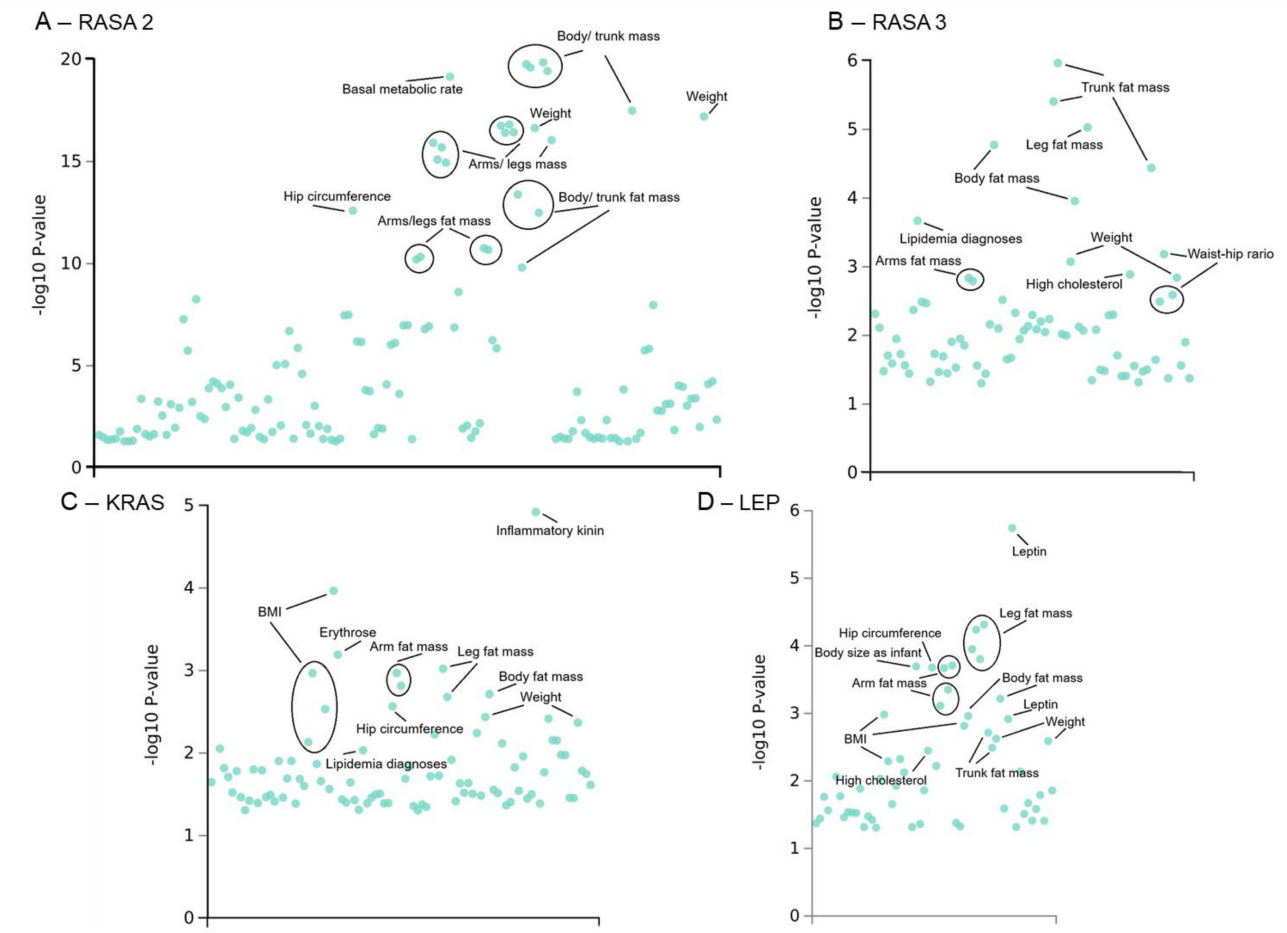
PheWAS associations for RASA2, RASA3, KRAS, and LEP across human metabolic phenotypes. PheWAS results from the GWAS Atlas showing the top phenotypic associations for RASA2 **(A)**, RASA3 **(B)**, KRAS **(C)**, and LEP **(D)**. Each point represents a single GWAS trait plotted by -log_10_(p-value). Labeled traits highlight the most significant clusters, which predominantly involve body fat mass, BMI, weight, basal metabolic rate, waist–hip ratio, lipidemia-related diagnoses, and regional fat depots (arms, legs, trunk). Additional biomarkers include inflammatory kinin (des-Arg bradykinin), erythrose, and circulating leptin. Panel-specific summary statistics: RASA2, 147 traits (Bonferroni-corrected threshold: 3.40×10^−4^); RASA3, 75 traits (threshold: 6.67×10^−4^); KRAS, 92 traits (threshold: 5.43×10^−4^).

Motivated by the conserved relationship between RasGAP1, Ras85D, and upd1 in *Drosophila* and their human orthologs RASA2/3, KRAS, and LEP, we sought to determine whether these human genes show comparable involvement in metabolic phenotypes, and therefore queried the GWAS Atlas and performed a systematic PheWAS analysis (*see Methods*). Notably, when investigating outputs related to metabolic and anthropometric phenotypes, a clear pattern emerged in which the most significant associations for all four genes clustered around obesity-related traits, including body fat mass, BMI, weight, and lipid metabolism (**Figure 5**).

For RASA2 (**Figure 5A**), the strongest associations were clustered around body and trunk mass, regional fat mass (arms and legs), weight, basal metabolic rate, and hip circumference. RASA3 showed a similar pattern (**Figure 5B**), with prominent associations for body fat mass, trunk and leg fat mass, weight, waist-hip ratio, and lipidemia phenotypes. KRAS displayed a broad metabolic footprint (**Figure 5C**), including associations with BMI, arm and leg fat mass, hip circumference, body fat mass, and lipid disorders, as well as biomarkers such as erythrose and des-Arg bradykinin (inflammatory kinin). As expected, LEP was strongly associated with circulating leptin levels, body fat mass, BMI, regional fat distribution, and high cholesterol.

Across all four genes, the PheWAS consistently highlighted fat mass, BMI, weight regulation, lipid metabolism, and central adiposity as the dominant phenotypic domains. This convergence supports the idea that the *Drosophila* RasGAP1–Ras85D–upd1 pathway has a conserved role in energy balance and adiposity, and that its human orthologs participate in metabolic trait and broad associations with obesity-related traits variation at the population level.

## Discussion

In this study, we combined cross-species comparative genomics, *Drosophila* behavioral and metabolic phenotyping, and human association-based analyses to implicate the RasGAP1-RASA2/3 axis as a candidate conserved regulator of energy balance. We show that *Drosophila* RasGAP1 is the closest evolutionary counterpart of mammalian RASA2 and RASA3, retaining a conserved multi-domain architecture and forming the invertebrate sister lineage to the vertebrate RASA2/3 clade. Functionally, pan-neuronal RasGAP1 knockdown produced a coordinated shift toward an obesity-like state, combining reduced locomotor output with increased feeding interactions and elevated triglycerides and circulating glucose. At the circuit–molecular interface, RasGAP1 suppression was associated with reduced expression of upd1, the *Drosophila* leptin analog (21), while interactome mapping and PheWAS analyses provided a translationally relevant context, linking Ras signaling and cytokine pathways.

Our evolutionary analyses provide a solid foundation for interpreting RasGAP1 phenotypes in a translational context. Prior *Drosophila* work on RasGAP1 (also termed Gap1) has focused primarily on development, where loss of RasGAP1 mimics constitutive Sevenless signaling and disrupts photoreceptor fate decisions (9), and where ectopic RasGAP1 expression suppresses receptor tyrosine kinase pathways and limits tissue growth (10, 11). These studies established RasGAP1 as a potent negative regulator of Ras-dependent signaling downstream of multiple growth factor receptors, but did not address its role in adult neural function or energy balance. Our work shifts the emphasis from developmental patterning to adult physiology, showing that neuronal RasGAP1 suppression is sufficient to reshape behavioral dimensions directly relevant to body-weight control.

The reduction in locomotor output together with increased feeding interactions suggests that RasGAP1 normally contributes to setting the balance between energy expenditure and consumption, rather than simply affecting one endpoint in isolation. Locomotor activity is a behavioral output related to the expenditure side of energy balance, and therefore should be interpreted as a complementary phenotype rather than a direct proxy for metabolic rate. Importantly, the reduction in locomotor output may reflect altered motivational state, altered metabolic state, or a secondary consequence of increased adiposity-like phenotype. The present data do not distinguish among these possibilities.

A key question raised by the behavioral phenotype is how a neuronal RasGAP can influence systemic energy balance. In *Drosophila*, metabolic control is strongly shaped by neuroendocrine and neuromodulatory circuits that couple sensory and internal-state information to hormonal and behavioral outputs (35). Ras signaling has established links to metabolic regulation in flies through its ability to interface with insulin/PI3K and MAPK/ERK pathways. For example, direct input from Ras to PI3K is required for maximal nutrient- and insulin-responsive PI3K signaling *in vivo*, with physiological consequences for growth and metabolic output (36). Separately, MAPK/ERK signaling has been shown to tune insulin sensitivity by regulating insulin receptor expression, thereby maintaining appropriate circulating glucose levels (37). Although these studies interrogate Ras pathway activity broadly rather than RasGAP1 specifically, they provide a mechanistic background in which altering Ras inactivation dynamics could plausibly shift nutrient-responsive signaling thresholds within neuroendocrine pathways.

Our findings that pan-neuronal RasGAP1 knockdown increases feeding interactions alongside elevated circulating sugars and triglycerides point to an impaired neuroendocrine coordination of energy balance. In *Drosophila*, this coordination is executed by defined brain circuits that integrate internal state with endocrine-like outputs to tune feeding, nutrient allocation, and storage (35). We turned our investigation to the particularly compelling candidate pathway involving the leptin-like satiety system, which is functionally conserved despite species differences in hormone source and anatomy (22, 38). In flies, the leptin analog Unpaired-1 (upd1) is produced in the brain and acts through the Domeless (dome) receptor on neuropeptide-F neurons to suppress attraction to food cues, feeding drive, and weight gain (21). In fact, disruption of this brain-derived leptin-analog circuit is sufficient to produce multiple hallmarks of obesity, supporting an evolutionarily conserved mechanism for central satiety control (21). Our qPCR data showing reduced upd1 expression in RasGAP1 knockdown flies provide candidate evidence linking Ras/Rap signaling to this canonical satiety pathway. However, because our approach was candidate-based rather than transcriptome-wide, the present data do not establish upd1 as the sole or primary downstream effector of RasGAP1. The accompanying *Drosophila* STRING network further supports this connection by placing RasGAP1 and upd1 within a coherent interaction pathway that includes Ras85D, dome, and additional cytokine/growth-factor-linked components (e.g., upd3, Krn, sty). While STRING does not establish directionality, the organization is notable as it positions Ras signaling regulators in close proximity to JAK/STAT-linked ligands and receptors that shape feeding-related circuit state in mammals (39), providing a plausible network substrate through which altered Ras signaling dynamics could bias leptin-analog satiety signaling.

The cross-species picture becomes even more compelling when the fly network is projected onto human ortholog space. In humans, the ortholog set RASA2/3–KRAS–LEP forms a connected neighborhood in STRING, and the presence of KRAS is conceptually important as it sits at the intersection of growth-factor signaling and metabolic context. Indeed, KRAS-driven signaling has been explicitly discussed as being modulated by obesogenic environments through inputs such as insulin resistance, inflammation, and gut-derived factors (40, 41). While most of the literature connecting KRAS and obesity comes from cancer biology, where KRAS mutations are key drivers of pancreatic tumorigenesis (40), strong evidence is provided for its broader relevance to energy homeostasis. In particular, KRAS-centered networks are sensitive to metabolic state and can engage feedback-rich signaling architectures that couple nutrient state to downstream cellular programs (41). In parallel, our PheWAS analysis in GWAS Atlas shows convergent enrichment of RASA2, RASA3, KRAS, and LEP across obesity-linked phenotypic domains, with dominant associations spanning fat mass, BMI, regional adiposity, and lipid dysregulation. These findings align with prior human observations that RASA3 methylation is associated with dietary and adiposity-related traits (5), and with emerging work positioning RASA2/3 as gatekeepers that modulate immune-metabolic signaling (3). Taken together, the fly behavioral and metabolic outcomes, the cross-species protein interactome maps, and the human phenome-wide signal converge on the idea that RasGAP proteins participate in a conserved signaling pathway that interfaces with leptin-like satiety control and adiposity-related physiology. However, these analyses should be viewed as translationally contextualizing and hypothesis-generating, not mechanistically definitive.

Several limitations of the present study should be acknowledged explicitly. First, the phenotypes reported here rely on a single pan-neuronal RNAi strategy, and orthogonal genetic validation using an additional RNAi line, rescue design, or independent allele would strengthen confidence in specificity. We note, however, that whole-body knockdown would be difficult to interpret for the present question because RasGAP1 has established developmental roles in multiple tissues, whereas our aim here was specifically to isolate the neuronal contribution. Second, the knockdown was constitutive from development onward, so developmental and adult neuronal effects cannot be dissociated in the current design; an inducible adult-specific system such as GS-elav would be a useful future refinement. Third, not all phenotypes were equally robust across both parental controls. The TAG and glucose phenotypes were stronger, whereas activity and trehalose measures require more cautious interpretation. Fourth, the metabolic analyses were designed as whole-organism readouts; they do not localize lipid accumulation to specific tissues. Finally, the link to the leptin-analog pathway remains preliminary, as we measured transcript abundance of upd1 relative to the driver control, but did not test the UAS-alone control in qPCR, did not assess Upd1 protein levels, upd2/upd3 or dome expression, did not resolve cell autonomy, and did not perform rescue/epistasis experiments. Accordingly, the qPCR result does not independently establish the RasGAP1–upd1 relationship, but rather provides a candidate observation consistent with the broader behavioral and metabolic phenotype.

Various next steps can follow directly from these results. Although experimental interrogation of Ras85D/KRAS was beyond the scope of the current work, our data nominate Ras85D as a high-priority intermediary linking RasGAP1 perturbation to leptin-analog circuitry. In *Drosophila*, Ras85D (also termed Ras1) is the canonical Ras GTPase that functions as a core component of receptor tyrosine kinase signaling, most prominently in the EGFR to Raf/MEK/ERK cascade, and it has been extensively implicated in developmental programs controlling growth, cell-cycle progression, and fate specification (42–44). Moreover, Ras85D can also contribute to energy-related signaling output, including contexts in which Ras input is required to achieve maximal PI3K signaling, linking Ras activity to pathways that regulate growth and metabolism (36). Despite this mechanistic foundation, the possible roles of Ras85D in the context of adult regulation of behavioral and metabolic states have not been yet investigated. This gap makes Ras85D a particularly tractable target for follow-up experiments. For example, manipulating Ras85D activity or downstream Raf/MEK/ERK effectors in the RasGAP1 knockdown background would directly test whether Ras85D-dependent signaling is necessary or sufficient for the behavioral and metabolic phenotypes. Likewise, Upd1 overexpression or related rescue/epistasis experiments represent an important next step to assess whether the behavioral and metabolic phenotypes can be functionally dissociated or rescued through this candidate satiety pathway. Finally, translation-oriented work could move beyond association by testing whether perturbing RASA2/3 in mammalian appetite circuits alters feeding or metabolic phenotypes, and whether these effects intersect with leptin signaling sensitivity or downstream KRAS pathway engagement. Together, these approaches would turn the present network-informed framework into a causal map, clarifying where RasGAP control enters satiety circuitry and how that control propagates to organism-level energy balance.

In conclusion, RasGAP1 emerges from this work as a candidate neuro-metabolic regulator that links intracellular Ras signaling control to organism-level energy balance. Neuronal RasGAP1 suppression shifts behavior and physiology in a coordinated direction, with increased feeding interactions, reduced locomotor output, and a systemic metabolic profile consistent with enhanced fuel availability and storage. Reduced upd1 expression associates this phenotype with a central leptin-analog pathway that modulates feeding in flies, and interactome mapping places RasGAP1 in a signaling neighborhood where Ras and cytokine pathways converge, nominating Ras85D as a plausible intermediary. Human network and phenome wide association signals further point to the same axis, with RASA2/3, KRAS, and LEP consistently enriched in adiposity and lipid related traits. In sum, our work provides evidence that variation in RasGAP1-RASA2/3 function can bias obesity risk by subtly reprogramming feeding-control circuitry, offering a plausible mechanistic basis for adiposity associations observed in human cohorts.

## Data Availability

The publicly available datasets analyzed in this study can be found in online repositories, with repository names and accession number(s) provided in the article and/or **Supplementary Material**. The original Drosophila data generated for this study are included in the article and **Supplementary Material**. Further inquiries can be directed to the corresponding author(s).
